# Redefining Plant-Necrotroph Interactions: The Thin Line Between Hemibiotrophs and Necrotrophs

**DOI:** 10.3389/fmicb.2021.673518

**Published:** 2021-04-28

**Authors:** Sivasubramanian Rajarammohan

**Affiliations:** Agricultural Biotechnology Division, National Agri-Food Biotechnology Institute, Mohali, India

**Keywords:** necrotroph, hemibiotroph, *Alternaria*, *Botrytis*, *Sclerotinia*

## Introduction

Fungal pathogens are a heterogeneous group of organisms which differ in many important traits such as mode of nutrition, type of reproduction, and dispersal mechanisms. Traditional classification of fungal pathogens has relied on their mode of nutrition to classify them into three broad categories viz. biotrophs, necrotrophs, and hemibiotrophs. Biotrophs derive nutrients and energy from living cells, while necrotrophs derive their energy from dead or dying cells. Hemibiotrophs initially invade live cells prior to transitioning to a necrotrophic lifestyle to obtain nutrients from killing the host cells. Necrotrophic pathogens have traditionally been perceived as “unsophisticated brutes” that employ a “Kill and feed” approach with the use of a battery of secreted toxins and cell-wall degrading enzymes (CWDEs). However, the perception of necrotrophic pathogens has undergone rapid changes in the last two decades. The experimental evidences that has changed the definition of a typical necrotrophic lifestyle are discussed below. Further, I propose that older definitions and classification of necrotrophs should be revisited in light of these evidences.

## Presence of A “Cryptic” Biotrophic Phase in Necrotrophic Lifestyles

*Botrytis cinerea* and *Sclerotinia sclerotiorum* are considered archetypal necrotrophs that have a broad host-range and infect most dicots. Though initial studies attributed their indiscriminate necrotrophic lifestyle to secretion of various CWDEs, oxalic acid, and toxins; recent studies have shown very intricate mechanisms underlying their pathogenesis. For example, *S. sclerotiorum* has been shown to dynamically modulate the host cellular environment (using oxalic acid) and suppress host defenses and cell death initiation in the early stages of infection (Williams et al., 2011). Furthermore, *S. sclerotiorum* hyphae grow intercellularly without causing cell death during early infection. This biotrophic growth of the hyphae coincides with the suppression of host cell death by oxalic acid. The leading edge of the lesion consists of biotrophic hyphal growth with cell death lagging behind (Kabbage et al., 2015). Recently, a two-phase infection model was proposed in which the pathogen evades and subverts the host defenses and later proceeds to killing the host cells (Liang and Rollins, 2018). Similarly, for *B. cinerea*, studies have shown that it can grow asymptomatically within the host before switching to a necrotrophic lifestyle. Multiple *Botrytis* species are capable of asymptomatic growth within the host and switch to necrotrophic development under undefined conditions (van Kan et al., 2014).

Cytological studies of *A. brassicae*, a necrotroph that infects most Brassicas, also reveal a similar pattern of a biotrophic phase. The advancing infection front had no associated cell death despite a large amount of intercellular hyphal growth. Also, cell death and reactive oxygen species (ROS) production was suppressed at the earlier stages of infection of *A. brassicae* (Mandal et al., 2018). The findings from *A. brassicae* is significant, since *Alternaria* genus is one of the prototypical toxin-producing genera, that have been implicated to cause cell death and infection utilizing host-specific and non-specific toxins.

The presence of a silent or cryptic biotrophic phase in necrotrophs has also been previously described in the context of postharvest pathogens wherein they are referred to as a quiescent stage. Quiescence refers to an extended period of time during which the pathogen activity is suspended, and no apparent growth takes place. Although quiescence is defined as involving no pathogen growth, pathogens often are actively involved in suppressing host defenses and hyphal growth is also observed in many cases. This phase in the infection cycle is referred to as biotrophic-quiescence. Several species of fungal pathogens, such as *Colletotrichum, Alternaria, Botrytis, Monilinia, Sclerotinia*, and *Botryospheria*, are known to quiescently live in the hosts and trigger necrosis at later stages of the infection (Prusky, 1996; Adaskaveg et al., 2000). Therefore, the presence of a “cryptic” biotrophic phase in many necrotrophs seems to be the rule rather than an exception.

## Necrotrophic Effectors—Determinants of Host Response Suppression?

Effectors are secreted by various pathogens to modulate host responses to the pathogen. Though most initial studies identified and characterized effectors from biotrophic and hemibiotrophic pathogens due to their lifestyle of suppressing host cell death, genome sequencing of various necrotrophs have revealed that necrotrophs also encode and secrete a number of effectors (Derbyshire et al., 2017; Van Kan et al., 2017; Rajarammohan et al., 2019). Functional studies of various effectors in *S. sclerotiorum* reconfirm the two-phased infection model. Effectors that support a biotrophic lifestyle, like Sscmu1 (chorismate mutase), LysM (chitin binding proteins), and ROS scavengers are upregulated and secreted in the early infection stages of *S. sclerotiorum*. Furthermore, the necrosis inducing effectors are upregulated in the later stages or downregulated in the early stages (Chittem et al., 2020). The necrotrophic effectors have been found to suppress and destabilize host immunity by targeting plant organelles such as chloroplast and mitochondria (Lyu et al., 2016; Tang et al., 2020). Transcriptome and proteome studies of the archetypal necrotrophs reveal that the CWDE activity of these pathogens are only activated in the later stages of infection and are generally suppressed during the early stages of infection (Peng et al., 2017; Westrick et al., 2019; Chittem et al., 2020). Therefore, similar to hemibiotrophic pathogens, these necrotrophic pathogens also temporally regulate the secretion of effectors and CWDEs to initially suppress host responses and establish themselves in the host.

## The Line Differentiating Necrotrophs and Hemibiotrophs—Thin or Non-Existent?

**Figure 1:**
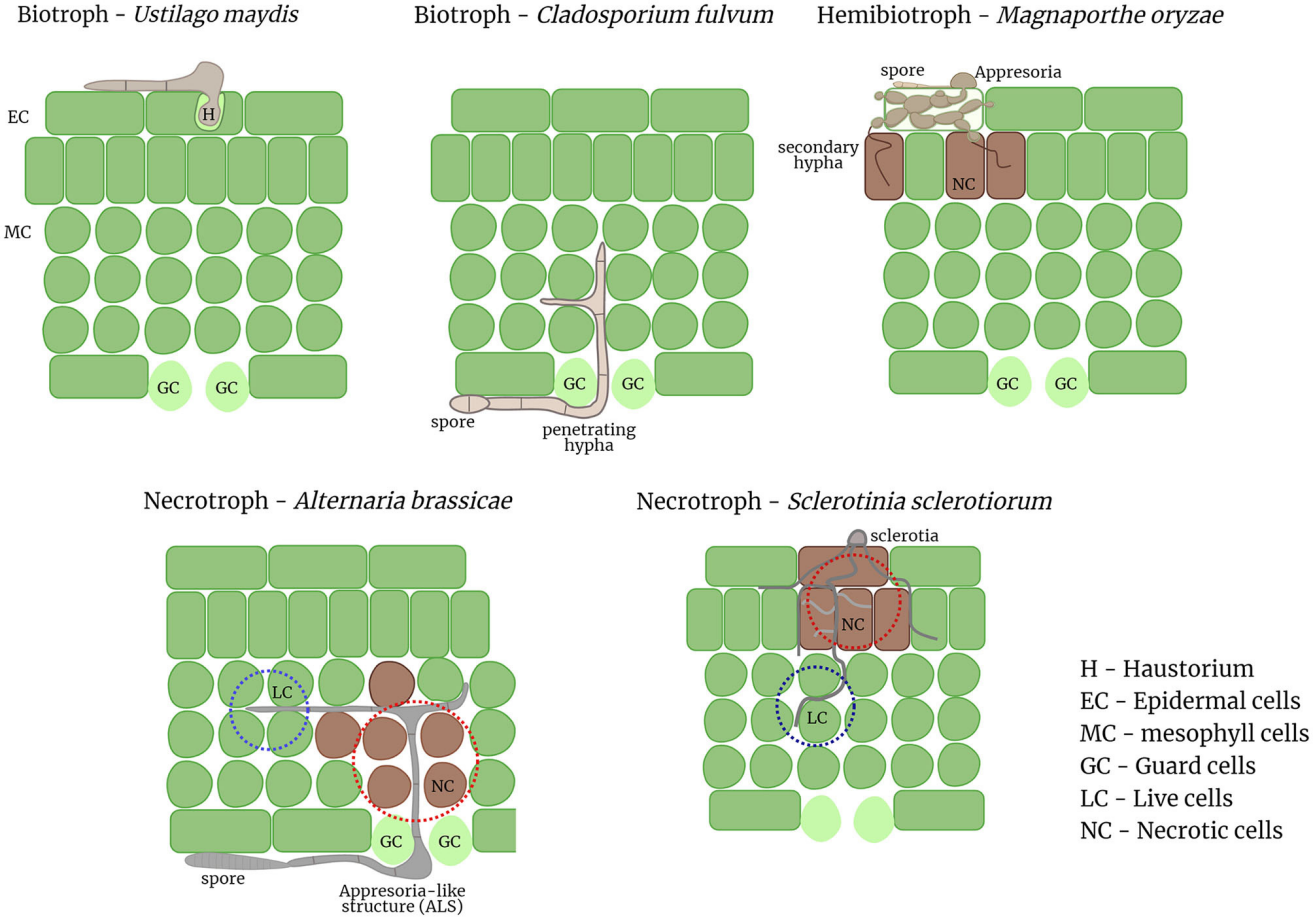
Pathogenic invasion strategies of diverse pathogens from different trophic lifestyles. Biotrophic pathogens like *U. maydis* form a haustorium inside a host cell to acquire nutrients from the host while biotrophs like *C. fulvum* grow extracellularly between the host cells. Hemibiotrophs like *M. oryzae* form penetration pegs called appresoria to invade the host cell; and switch to necrotrophy later when secondary hyphae are formed. Necrotrophs like *A. brassicae* invade primarily through the stomata by forming ALS whereas *S. sclerotiorum* invades the host cells directly; the leading edge of the hyphae, in both necrotrophs grows intercellularly between the host cells similar to the “biotrophic phase” of hemibiotrophs. Necrosis is often induced in the trailing host cells where secondary hyphae start to emerge.

There is growing evidence from various cytological, genomics, and functional studies for multiple necrotrophic pathogens of different genera suggestive of a biotrophic phase prior to the onset of cell death. In light of these emerging evidences the factors that can clearly differentiate between hemibiotrophs and necrotrophs are next to none. Both hemibiotrophic and necrotrophic lifestyles consist of an initial biotrophic phase followed by necrosis. The duration of the biotrophic phase varies even within hemibiotrophs and may vary even more in the case of necrotrophs and may be influenced by a myriad of external environmental factors. The necrotrophs discussed herein all form appresoria or appresoria-like structures (ALS), and in some cases bulbous hyphae as in case of hemibiotrophs. One of the major differential feature between hemibiotrophs and these necrotrophs is the formation of haustoria or haustoria-like structures from intracellular hyphae, which the hemibiotrophs share with the biotrophic pathogens. Most hemibiotrophs initially form an intracellular bulged hypha that is encased by the host plasma membrane and later switch to a thinner intracellular hypha that initiates necrosis (Giraldo and Valent, 2013). In the necrotrophs, no intracellular growth of hyphae is observed, but the hyphae grow extracellularly and penetrates through stomatal openings or hydathodes. However, the biotrophic phase of these necrotrophs are characterized by extracellular or intercellular hyphal growth, which is reminiscent of the biotrophic fungal pathogen, *Cladosporium fulvum*. *C. fulvum* grows extracellularly and enters the host through the stomata. Therefore, the biotrophic phase of necrotrophs can be deemed similar to the hyphal growth non-haustoria forming biotrophic pathogens (Figure 1).

Given the substantial similarities between the lifestyles of hemibiotrophs and necrotrophs, I postulate that necrotrophs that display a two-phase infection process and share most features of hemibiotrophs be classified as “extracellular” hemibiotrophs. The reclassification is important since it highlights the presence of a biotrophic phase in these pathogens and the fact that these necrotrophs secrete various effectors during this early stage to suppress host cell death. The effectors secreted by necrotrophic pathogens during the biotrophic phase may share similarity (sequence/structural/functional) with biotrophic effectors that are recognized by the host surveillance system (R-genes) to mount a defense response. Therefore, identification and characterisation of these early stage effectors may reinvigorate the search for the elusive gene-for-gene resistance against these necrotrophic pathogens.

## Conclusions

The demarcation between the different trophic lifestyles has turned out to be an artifact created by our imperfect understanding of biological systems. Necrotrophic fungal pathogens are complex and their interactions with the host are much more nuanced than previously thought. The definition of necrotrophs as brute force killers has precluded the study of effectors and gene-for-gene interactions in their respective hosts. With the advent of genome sequencing studies, effectors have been identified in necrotrophs and are known to play a role in suppressing host responses including host cell death. The presence of an early biotrophic phase, effectors that suppress and modulate host responses, presence of appresoria or ALS, temporal and spatial regulation of toxins and CWDEs in some of the necrotrophic pathogens support the redefinition or renaming of these necrotrophs as “extracellular” hemibiotrophs. Additionally, cytological studies of early stages of infection of other necrotrophs needs to be revisited in the light of the recent findings to gain better understanding of the necrotrophic lifestyle and redefine their lifestyle if similar features are discovered. I believe that the redefinition of necrotrophs exhibiting the above described features will help illuminate the need to characterize early stage effectors to improve our understanding of necrotrophic pathogens and provide the basis for identifying the complementary host resistance components.

## Author Contributions

SR conceived the idea and wrote the manuscript.

## Conflict of Interest

The author declares that the research was conducted in the absence of any commercial or financial relationships that could be construed as a potential conflict of interest.

## References

[B1] Adaskaveg J. E. Forster H. Thompson D. F. (2000). Identification and etiology of visible quiescent infections of *Monilinia fructicola* and *Botrytis cinerea* in sweet cherry fruit. Plant. Dis. 84, 328–333. 10.1094/PDIS.2000.84.3.328 30841251

[B2] Chittem K. Yajima W. R. Goswami R. S. Del Rio Mendoza L. E. (2020). Transcriptome analysis of the plant pathogen *Sclerotinia sclerotiorum* interaction with resistant and susceptible canola (*Brassica napus*) lines. PLoS ONE 15:e0229844. 10.1371/journal.pone.0229844 32160211 PMC7065775

[B3] Derbyshire M. Denton-Giles M. Hegedus D. Seifbarghy S. Rollins J. van Kan J.. (2017). The complete genome sequence of the phytopathogenic fungus *Sclerotinia sclerotiorum* reveals insights into the genome architecture of broad host range pathogens. Genome Biol. Evol. 9, 593–618. 10.1093/gbe/evx030 28204478 PMC5381539

[B4] Giraldo M. C. Valent B. (2013). Filamentous plant pathogen effectors in action. Nat. Rev. Microbiol. 11, 800–814. 10.1038/nrmicro3119 24129511

[B5] Kabbage M. Yarden O. Dickman M. B. (2015). Pathogenic attributes of *Sclerotinia sclerotiorum*: switching from a biotrophic to necrotrophic lifestyle. Plant. Sci. 233, 53–60. 10.1016/j.plantsci.2014.12.018 25711813

[B6] Liang X. Rollins J. A. (2018). Mechanisms of broad host range necrotrophic pathogenesis in *Sclerotinia sclerotiorum*. Phytopathology 108, 1128–1140. 10.1094/PHYTO-06-18-0197-RVW 30048598

[B7] Lyu X. Shen C. Fu Y. Xie J. Jiang D. Li G.. (2016). A small secreted virulence-related protein is essential for the necrotrophic interactions of *Sclerotinia sclerotiorum* with its host plants. PLoS Pathog. 12:e1005435. 10.1371/journal.ppat.1005435 26828434 PMC4735494

[B8] Mandal S. Rajarammohan S. Kaur J. (2018). *Alternaria brassicae* interactions with the model *Brassicaceae* member *Arabidopsis thaliana* closely resembles those with Mustard (*Brassica juncea*). Physiol. Mol. Biol. Plants 24, 51–59. 10.1007/s12298-017-0486-z 29398838 PMC5787117

[B9] Peng Q. Xie Q. Chen F. Zhou X. Zhang W. Zhang J.. (2017). Transcriptome analysis of *Sclerotinia sclerotiorum* at different infection stages on *Brassica napus*. Curr. Microbiol. 74, 1237–1245. 10.1007/s00284-017-1309-8 28785831

[B10] Prusky D. (1996). Pathogen quiescence in postharvest diseases. Ann. Rev. Phytopathol. 34, 413–434. 10.1146/annurev.phyto.34.1.413 15012550

[B11] Rajarammohan S. Paritosh K. Pental D. Kaur J. (2019). Comparative genomics of *Alternaria* species provides insights into the pathogenic lifestyle of *Alternaria brassicae*- a pathogen of the *Brassicaceae* family. BMC Genom. 20:1036. 10.1186/s12864-019-6414-6 31888481 PMC6937934

[B12] Tang L. Yang G. Ming Y. Xiaofan M. Bo L. Jiatao L.. (2020). An effector of a necrotrophic fungal pathogen targets the calcium sensing receptor in chloroplasts to inhibit host resistance. Mol. Plant. Pathol. 21, 686–701. 10.1111/mpp.12922 32105402 PMC7170781

[B13] van Kan J. A. Shaw M. W. Grant-Downton R. T. (2014). *Botrytis species*: relentless necrotrophic thugs or endophytes gone rogue? Mol. Plant. Pathol. 15, 957–961. 10.1111/mpp.12148 24754470 PMC6638755

[B14] Van Kan J. A. Stassen J. H. Mosbach A. Van Der Lee T. A. Faino L. Farmer A. D.. (2017). A gapless genome sequence of the fungus *Botrytis cinerea*. Mol. Plant. Pathol. 18, 75–89. 10.1111/mpp.12384 26913498 PMC6638203

[B15] Westrick N. M. Ranjan A. Jain S. Grau C. R. Smith D. L. Kabbage M. (2019). Gene regulation of *Sclerotinia sclerotiorum* during infection of *Glycine max*: on the road to pathogenesis. BMC Genom. 20:157. 10.1186/s12864-019-5517-4 30808300 PMC6390599

[B16] Williams B. Kabbage M. Kim H. J. Britt R. Dickman M. B. (2011). Tipping the balance: *Sclerotinia sclerotiorum* secreted oxalic acid suppresses host defenses by manipulating the host redox environment. PLoS Pathog. 7:e1002107. 10.1371/journal.ppat.1002107 21738471 PMC3128121

